# Changes in diet and physical activity in adolescents with and without type 1 diabetes over time

**DOI:** 10.1186/1687-9856-2014-17

**Published:** 2014-08-15

**Authors:** Franziska K Bishop, R Paul Wadwa, Janet Snell-Bergeon, Nhung Nguyen, David M Maahs

**Affiliations:** 1 Barbara Davis Center for Childhood Diabetes, University of Colorado Denver, 1775 Aurora Ct, MS F527, 80045 Aurora, CO, USA

**Keywords:** Diet, Physical activity, Type 1 diabetes, Screen time, Adolescents

## Abstract

**Background:**

Diet and physical activity (PA) are fundamental aspects of care in type 1 diabetes, but scant longitudinal data exist on these behaviors in adolescents with type 1 diabetes, especially compared to non-diabetic controls.

**Methods:**

Data in 211 adolescents with type 1 diabetes (baseline age = 15.3 ± 2.2 years, diabetes duration = 8.8 ± 3.1 years, A1c = 9.0 ± 1.5%, 51% male) and 67 non-diabetic (age = 14.9 ± 1.7 years, 52% male) controls were collected at baseline (V1) and again at 2-year follow-up (V2) (mean follow up = 2.2 ± 0.4 years). Diet data (meals/day, snacks/day, and weekly consumption of breakfast, fruit, vegetables and fried foods), and PA were collected using interviewer administered questionnaires. T-tests and chi-squared tests were used for comparisons.

**Results:**

Both adolescents with type 1 diabetes and non-diabetic controls reported increased vegetable (2.8 v. 3.6 and 3.1 v. 3.8 times weekly, respectively, p < 0.0001) and fruit (2.9 v. 3.8, both groups, p < 0.0001) intake (times per week) and increased PA (hours/day; 1.8 v. 2.2, p = 0.005 and 1.5 v. 1.9, p = 0.008, respectively) from V1 to V2. Adolescents with type 1 diabetes reported eating breakfast (3.3 v. 3.8 weekly, p = 0.0002) but also fried foods (1.9 v. 2.3, p = 0.0005) weekly more often from V1 to V2. Adolescents with and without type 1 diabetes met PA recommendations of 60 minutes or more of moderate-to-hard PA daily at both V1 (74% v. 70%, respectively, p = 0.58) and V2 (70% v. 78%, respectively, p = 0.78).

**Conclusions:**

Over 2 years, adolescents with and without type 1 diabetes had a healthier diet with increased fruit and vegetable intake and increased PA. However, neither group met the guidelines of daily breakfast, fruit and vegetable intake. Some diet and PA improvements were seen in adolescents with type 1 diabetes over a 2-year period. Therefore, adolescence could be a beneficial time to target diet and lifestyle interventions to take advantage of this time period when behaviors are being modified.

## Background

Diet and physical activity (PA) are fundamental aspects of care in type 1 diabetes, but scant data exist on diet and PA behaviors of adolescents with type 1 diabetes, especially compared to non-diabetic (non-DM) controls [1]. The nutrition guidelines for children with type 1 diabetes are similar to those for the general population with a focus on healthful eating to provide energy intake and nutrients to ensure normal growth and development [2-4]. The USDA Guidelines for Americans 2010 recommends to “build a healthy plate” with half a plate of fruits and vegetables; cut back on foods high in saturated fat, added sugar and salt; choose whole grains and be physically active [5]. The American Academy of Pediatrics (AAP) recommends eating 3 balanced meals/day, eating breakfast daily, and limiting sugary beverages and energy dense foods [6]. The American Diabetes Association (ADA) recommends a diet that includes carbohydrates from fruits, vegetables, and whole grains and limit saturated fat and cholesterol [4].

The SEARCH for Diabetes in Youth study reported that adolescents with type 1 diabetes do not meet the ADA and AAP dietary recommendations in cross-sectional data [7]. Similarly, youth without diabetes also do not meet the U.S. dietary guidelines [8,9]. Adolescents with and without diabetes do not consume adequate fruit and vegetables and consume too much saturated fat [7,10,11]. However, few studies have compared dietary patterns in adolescents with type 1 diabetes to non-DM controls, and none longitudinally [1].

Youth with type 1 diabetes are encouraged to exercise for health to promote benefits such as weight control, cardiovascular fitness, improved blood pressure, and lipid profile [12]. The ADA recommends moderate-intensity PA for at least 30-60 minutes per day [12] and limiting sedentary activities. Current PA guidelines recommend that all school-age children participate in at least 60 minutes of moderate-to-vigorous intensity daily [13] and the AAP recommends no more than 1-2 hours of screen time per day [14]. Excessive screen time can replace or limit PA and has been linked to obesity, poorer glycemic control, behavioral problems, and impaired academic performance [14,15].

Herein we report diet, PA and screen time patterns in youth with and without type 1 diabetes in the “Determinants of Macrovascular Disease in Adolescents with Type 1 Diabetes” study at baseline and at the 2-year follow-up visit. Our objective is to examine the differences in diet and PA in adolescents with and without type 1 diabetes over 2 years. Diet and PA recommendations are similar for adolescents with and without type 1 diabetes; however, adolescents with type 1 diabetes receive regular care from their diabetes care team and should receive more diet and PA education, support and counseling. Therefore, we hypothesized that adolescents with type 1 diabetes would have improved diet and PA patterns compared to non-DM controls at baseline and over the course of the study period.

## Methods

The Determinants of Macrovascular Disease in Adolescents with Type 1 Diabetes study prospectively assessed early cardiovascular disease (CVD) risk factors in youth with and without type 1 diabetes [16-18]. The main specific aim was to assess the prevalence, treatment, and control of established CVD risk factors, including glycemic control, blood pressure, and lipid levels, and their association with vascular function in a cohort of 300 youth with type 1 diabetes, age 12-19 years. Secondly, the study compared the CVD risk profile of the 300 youth with type 1 diabetes with that of 100 non-DM controls of similar age and gender. The study then followed the cohort for 2 years. Enrollment began in 2008 for the baseline visit (V1) and concluded in 2012 for the 2-year follow-up visit (V2). At V1, participants were ages 12-19 and those with type 1 diabetes were diagnosed by the presence of islet antibodies or by provider clinical diagnosis, had diabetes duration > 5 years at entry into the study, and received care at the Barbara Davis Center for Childhood Diabetes. Non-DM control participants were recruited from campus and community advertisements as well as from friends of the study participants. No siblings of patients with type 1 diabetes were included. Participants were excluded for diabetes of any other type. The study was approved by the Colorado Multiple Institution Review Board and informed consent and assent (for subjects <18 years) was obtained in all participants. All study participants were invited back for V2.

At V1 and V2 all participants had anthropometric and questionnaire data collected. Diet data (meals/day, snacks/day, and weekly consumption of breakfast, fruit, vegetables, sweets/desserts, fried foods, restaurant meals, and sugary beverages), physical activity, and screen time (T.V., video games, and computer) were collected using interviewer-administered questionnaires (Additional file 1). Adolescents were administered the questionnaire alone without parent(s) in the room. Dietary practices consisted of frequency of consumption of the following: restaurant meals (including fast food), sweetened beverages, meals per day, snacks per day, breakfast, fruit, vegetables, fried foods and sweets/desserts. Frequency was coded as times per week. Meals per day and snacks per day were reported as: one, two, three, four or five or more per day. For PA, days per week, hours per day (on days where PA took place) and exercise level (light, moderate and hard; the definition of intensity was explained to participants with examples) were asked. Meeting PA guidelines was defined as 60 minutes or more of moderate-to-hard physical activity daily per U.S and international PA recommendations. For screen time; hours per day on TV, hours per day on computer and hours per day on video/electronic games were asked. For total screen time, TV, computer and video/electric games hours per day were combined. Height was measured by a wall-mounted stadiometer to the nearest 0.1 cm with shoes removed and weight by a Detecto scale to the nearest 0.1 kg. Waist circumference was measured at the navel on bare skin using the Figure Finder Tape Measure by Novel Products, Inc (Rockton, IL), which provides consistent and repeatable 4 oz. of tension and accuracy to 3/32 inch. BMI was calculated and in subjects <20 years of age, BMI z-score and BMI percentile was calculated using the 2000 Centers for Disease Control and Prevention growth chart standards. When calculating BMI-z-score and BMI-percentile at V2, 42 participants were excluded from the analysis due to age cutoffs (>20 years old). HbA1c was measured on the DCA Advantage by Siemens at the Children’s Hospital Colorado (Aurora, CO) main clinical lab.

### Statistical analysis

Stratified two sample t-tests were used to examine differences across groups for continuous variables, and chi square tests were used to test for differences in categorical data. Change between V1 and V2 in each group was measured using paired t-tests. Only participants that completed both V1 and V2 were included (N = 278). Pearson correlations were performed to calculate correlation coefficients for BMI-z and HbA1c with dietary habits, PA, and screen time for T1D and non-DM. Multivariate analyses were used to determine the effect of sex on dietary habits, PA, and screen time. A p-value less than 0.05 was considered significant. Analyses were performed using SAS version 9.3 (SAS Institute, Cary, NC).

## Results

A total of 400 participants completed V1 (type 1 diabetes n = 300, non-DM n = 100) and 278 participants completed V2 (type 1 diabetes n = 211, non-DM n = 67). Data in 211 adolescents with type 1 diabetes (baseline age = 15.3 ± 2.2 years, diabetes duration = 8.8 ± 3.1 years, A1c = 9.0 ± 1.5%, 51% male) and 67 non-DM controls (age = 14.9 ± 1.7 years, 52% male) were collected at V1 and again at V2 (mean follow up = 2.2 ± 0.4 years) and were included for this analysis (Table 1). Age and gender were similar in both groups at V1 and V2, but race-ethnicity differed, and adolescents with type 1 diabetes were slightly older than non-DM controls at V2. Adolescents with type 1 diabetes had higher BMI (p = 0.05) and BMI z-score (p = 0.01) at V1; there were no differences between diabetes status and BMI percentile categories (normal, <85^th^ percentile and overweight or obese, ≥85^th^ percentile) at V1. At V2, BMI z-score was significantly higher in adolescents with type 1 diabetes compared to non-DM controls (p = 0.04). There were no differences in BMI-percentile categories (normal, <85^th^ percentile vs. overweight or obese, >85^th^ percentile) in adolescents with type 1 diabetes compared to non-DM controls at V2. Waist and hip measurements were similar in both groups at both visits.

**Table 1:** Demographics at baseline (V1) and 2-year follow-up (V2)

	**Visit 1**			**Visit 2**		
	**T1D**	**Non-DM**	**p-value**	**T1D**	**Non-DM**	**p-value**
**Age, years**	15.3 ± 2.2	14.9 ± 1.7	0.13	17.5 ± 2.3	16.9 ± 1.9	0.04
**Male, %**	51%	52%	0.88	NA	NA	--
**Race-Ethnicity, % NHW**	86%	73%	0.01	NA	NA	--
**T1D Duration, years**	8.8 ± 3.1	NA	--	11.0 ± 3.2	NA	--
**CSII pump, %**	62%	NA	--	61%	NA	--
**HbA1c, %**	9.0 ± 1.5	5.3 ± 0.3	<0.0001	9.1 ± 1.7	5.2 ± 0.2	<0.0001
**BMI, kg/m**^**2**^	22.6 ± 3.7	21.4 ± 4.5	0.05	23.8 ± 3.6	22.8 ± 5.1	0.13
**BMI z-score**	0.61 ± 0.76	0.22 ± 1.1	0.01	0.55 ± 0.83	0.24 ± 1.05	0.04
**Waist, cm**	76.5 ± 9.9	74.4 ± 12.1	0.17	81.8 ± 9.3	78.7 ± 13.6	0.08
**Hip, cm**	94.0 ± 10.2	91.1 ± 11.1	0.06	97.7 ± 7.6	95.0 ± 11.2	0.07

T1D: Type 1 Diabetes.

Non-DM: non-diabetic control.

Table 2 shows the diet, PA, and screen time data for both groups at V1 and V2 and the change between visits within adolescents with type 1 diabetes and within non-DM controls. Both adolescents with and without type 1 diabetes reported increased vegetable and fruit intake from V1 to V2 and these changes were significant (all p-values <0.0001). Adolescents with type 1 diabetes reported eating breakfast more often from V1 to V2; however, at V2 both adolescents with type 1 diabetes and non-DM controls ate breakfast fewer than 4 times per week (3.8 vs. 3.7, respectively, p = 0.0002). Both adolescents with and without type 1 diabetes consumed 3 meals per day and this frequency was consistent from V1 to V2. Snack consumption was the same in both groups at approximately 2 snacks per day and this did not change from V1 to V2. Surprisingly, at V1 adolescents with type 1 diabetes consumed as many sugary beverages as non-DM controls (2.6 vs. 2.8 per week, respectively, p = 0.32); however, at V2 adolescents with type 1 diabetes consumed less sugary beverages (2.7 vs. 3.2, respectively, p = 0.02). For restaurant meals there was no difference in adolescents with and without type 1 diabetes at V1 or V2; however, adolescents with type 1 diabetes did eat significantly fewer restaurant meals at V2 than V1 (2.1 vs. 1.7, p = .0008). At V1, adolescents with and without type 1 diabetes consumed fried foods at the same frequency per week (1.9 v. 1.8, respectively, p = 0.51). At V2, adolescents with type 1 diabetes reported more fried food consumption (1.9 vs. 2.3, p = 0.0005) while there was no change in consumption for non-DM controls. Overall there was no difference in consumption of sweets/desserts among adolescents with and without type 1 diabetes with both groups consuming sweets/desserts twice a week. There were no significant changes in BMI-z score for adolescents with and without type 1 diabetes over time from V1 to V2 (Table 2).

**Table 2:** Change between baseline (V1) and 2-year follow-up (V2) within T1D and non-DM

	**T1D**			**Non-DM**		
	**Visit 1**	**Visit 2**	**Change**	**Visit 1**	**Visit 2**	**Change**
**Diet**						
Meals/day	3.0 ± 0.6	2.9 ± 0.6	-0.1 ± 7.5**	3.0 ± 0.7	2.9 ± 0.7	-0.06 ± 0.6
Snacks/day	2.3 ± 1.2	2.3 ± 1.3	-0.05 ± 1.3	2.2 ± 1.0	2.2 ± 1.1	-0.01 ± 1.2
Breakfast^#^	3.3 ± 1.2	3.8 ± 1.6	0.45 ± 1.7**	3.5 ± 1.0	3.7 ± 1.8	0.15 ± 1.7
Vegetables^#^	2.8 ± 1.1*	3.6 ± 1.4	0.87 ± 1.5**	3.1 ± 1.2*	3.8 ± 1.3	0.69 ± 1.3**
Fruit^#^	2.9 ± 1.2	3.8 ± 1.3	0.89 ± 1.4**	2.9 ± 1.3	3.8 ± 1.5	0.85 ± 1.6**
Sugary beverages^#^	2.6 ± 1.4	2.7 ± 1.7*	0.06 ± 2.1	2.8 ± 1.3	3.2 ± 1.7*	0.45 ± 2.2
Restaurant meals^#^	2.1 ± 1.6	1.7 ± 1.0	-0.44 ± 1.9**	2.2 ± 1.6	1.8 ± 1.2	-0.34 ± 2.3
Fried foods^#^	1.9 ± 1.2	2.3 ± 1.3	0.41 ± 1.7**	1.8 ± 1.1	2.1 ± 1.2	0.27 ± 1.6
Sweets/Desserts^#^	2.1 ± 1.3	2.2 ± 1.3	0.11 ± 1.7	2.3 ± 1.3	2.4 ± 1.7	0.18 ± 1.9
**Physical activity (PA)**						
PA days/week	5.0 ± 1.8	4.8 ± 1.7	-0.29 ± 2.2	4.9 ± 1.7	4.5 ± 1.9	-0.41 ± 2.3
PA hours/day	1.8 ± 1.2*	2.2 ± 1.4	0.35 ± 1.6**	1.5 ± 0.8*	1.9 ± 1.3	0.43 ± 1.2**
PA level^†^	1.9 ± 0.6	2.0 ± 0.5	0.06 ± 0.7	2.0 ± 0.5	2.1 ± 0.6	0.12 ± 0.7
**Screen time**						
Computer hours^+^	1.5 ± 1.7	2.5 ± 2.5	0.98 ± 2.6**	1.6 ± 1.7	2.2 ± 2.4	0.61 ± 1.9**
TV hours^+^	2.0 ± 1.9	1.9 ± 1.7	-0.12 ± 1.7	1.7 ± 1.6	1.9 ± 1.4	0.14 ± 1.5
Video games hours^+^	0.9 ± 1.3	0.8 ± 1.2	-0.08 ± 1.2	0.8 ± 1.1	0.7 ± 1.2	-0.04 ± 1.1
Total screen time^+^	4.3 ± 3.3	5.1 ± 3.5	0.78 ± 3.3**	4.1 ± 2.8	4.8 ± 3.0	0.7 ± 2.4**
**Anthropometric**						
BMI z-score	0.6 ± 0.8*	0.5 ± 0.8*	-0.04 ± 0.56	0.2 ± 1.1*	0.2 ± 1.1*	0.04 ± 0.46

T1D: Type 1 Diabetes.

Non-DM: non-diabetic control.

^#^Times per week.

^†^PA level: 1 = mild, 2 = moderate, 3 = hard.

^+^hours per day.

*p < 0.05 comparing T1D and non-DM at visit 1 or visit 2.

**p < 0.05 comparing change between visit 1 and visit 2 within T1D and non-DM.

At V1 74% of adolescents with type 1 diabetes met PA recommendations of 60 minutes or more of moderate-to-hard PA daily compared to 70% of non-DM controls (p = 0.58). At V2 the percentage of adolescents with and without type 1 diabetes that met PA recommendations increased to 80% and 78%, respectively, and the difference between groups was not significant (p = 0.78). Additionally, the change within adolescents with type 1 diabetes and within non-DM controls from V1 to V2 was not significant. Both groups performed PA on most days of the week at a moderate-to-hard level for at least 60 minutes on days PA was performed (see Table 2). At V1, adolescents with type 1 diabetes reported more time spent performing PA than non-DM controls (p = 0.02) yet the number of days of PA and level of intensity were the same in both groups. There were no differences reported in days per week PA was performed at V2 between groups, although both adolescents with and without type 1 diabetes reported an increase in PA time (hours per day on days PA was performed) from V1 to V2 (p = 0.005 and p = 0.008, respectively).

For screen time, TV and video/electronic game hours per day were the same for both adolescents with and without type 1 diabetes at V1 and V2 and there were no changes within each group from V1 to V2. Computer hours per day did increase for adolescents with type 1 diabetes and non-DM controls from V1 to V2 (1.5 vs. 2.5, p < 0.0001 and 1.6 vs. 2.2, p = 0.01, respectively). Total screen time increased to over 4 hours per day for adolescents with and without type 1 diabetes (p = 0.0009 and p = 0.02, respectively) due to the increased computer time reported.

In post-hoc analyses the associations between A1c and BMI-z score and diet, PA, and screen time in adolescents with type 1 diabetes were performed. At V1, there was a positive association with A1c and exercise (hours per day, p = 0.02) in adolescents with type 1 diabetes. At V2, A1c was positively correlated with screen time (TV hours, video game hours, overall screen time, p = 0.02, p = 0.02, p = 0.001, respectively) in adolescents with type 1 diabetes. BMI-z score was negatively correlated with meals per day (p = 0.001) and frequency of snacks (p = 0.04) at V2 in adolescents with type 1 diabetes.

## Discussion

Over 2 years, adolescents with and without type 1 diabetes reported a healthier diet with increased fruit and vegetable intake and increased PA. However, both groups are far from meeting the guidelines of daily fruit and vegetables intake or daily consumption of breakfast. Adolescents with type 1 diabetes also reported increased fried food intake from V1 to V2. This suggests that further attention to dietary recommendations and education are needed for adolescents with type 1 diabetes.

The SEARCH for Diabetes in Youth study also showed poor adherence to dietary guidelines with less than 50% of the cohort meeting ADA recommendations for total fat, fiber, fruits, vegetables and grains [7]. Although the SEARCH for Diabetes in Youth study used detailed food frequency methodology for diet data collection, the SEARCH data and our data similarly demonstrate that adolescents with type 1 diabetes did not meet the dietary guidelines of eating fruits and vegetables daily. Additionally, we were able to compare dietary patterns between adolescents with and without type 1 diabetes over time. Neither group met dietary recommendations despite adolescents with type 1 diabetes receiving dietary education from their diabetes care team.

In cross-sectional data, Nansel et al. reported that children and adolescents consume less than half of the recommended intake of fruit, vegetables, and whole grains and exceed saturated fat intake [19]. In addition, results from the Coronary Artery Calcification in Type 1 Diabetes (CACTI) study demonstrated that young adults with type 1 diabetes consume excess fat and saturated fat [20]. Similarly our data found that adolescents with type 1 diabetes are consuming more fried foods over time. A recent study in adolescents with type 1 diabetes reported that over half of youth with type 1 diabetes consume more cholesterol than recommended by U.S. and international guidelines and this was positively correlated with A1c and calories consumed as fat [21]. Poor dietary habits start in youth and can persist into adulthood, which is of particular concern for adolescents with type 1 diabetes due to their elevated risk of cardiovascular disease.

The majority of adolescents with type 1 diabetes (74%) and non-DM controls (70%) met the PA guidelines of 60 minutes or more of moderate-to-hard PA daily over the 2-year study period. Even though adolescents with type 1 diabetes reported increased PA time on active days compared to non-DM controls at V1, BMI and BMI z-score were higher in adolescents with type 1 diabetes. At V2, BMI z-score continued to be higher in adolescents with type 1 diabetes compared to non-DM controls. The T1D Exchange has recently reported that 39% of 13-19 year olds had a BMI > 85^th^% for age and sex [22]. The Hvidoere study group reported that adolescents with type 1 diabetes are only physically active for 60 minutes or more 3-4 days per week; therefore, not meeting international PA recommendations for 60 minutes of exercise daily [23] in contrast to our data. Overby et al. reported that only 54% of children and adolescents with type 1 diabetes meet PA recommendations [24]. NHANES data from 1999-2006 in U.S. adolescents aged 12-17 reported that less than 20% of adolescents reported meeting the 2008 Physical Activity Guidelines for Americans [25]. However, our cohort is from Colorado which is one of the healthiest states in the U.S. and ranked lowest for BMI and sedentary activity [26] and therefore may not be representative of the U.S. as a whole. The Youth and Risk Behavior Survey in 2011 reported that 53% of Colorado high school students reported physical activity at least 60 minutes per day on 5 or more days [27]. Additionally 23% of Colorado children are overweight or obese [28] with an obesity ranking of 23rd among the US states [29]. In our study 30% of adolescents with and without type 1 diabetes were overweight or obese. Even though this study’s participants meet the PA recommendations they are similarly overweight and obese compared to Colorado as a whole.

There may be sex-specific differences in PA; men with type 1 diabetes from the CACTI study reported increased PA than non-DM men but women with type 1 diabetes reported less PA than non-DM women [30]. The Hvidoere Study also found similar patterns in their adolescent cohort with males with type 1 diabetes being more active than females with type 1 diabetes [23]. These data suggest sex-specific differences in PA in adults and adolescents with type 1 diabetes. The current study found small differences between males and females with type 1 diabetes for time spent in PA at V1 or V2. Males with type 1 diabetes did report higher PA intensity at V2 (p = 0.047) than females with type 1 diabetes, and females reported more exercise days per week at V2 (p = 0.05) than males, but overall PA time was the same.

Screen time was elevated in both groups well over the 1-2 hour per day guidelines. Excess screen time is associated with many health and wellness issues such as obesity, higher A1c, poor academic performance, and decreased PA. Specifically, Overby et al. showed that children with type 1 diabetes watch an excess of amount of TV (>2 hours per day) and TV viewing was related to overweight children and adolescents with type 1 diabetes [24]. Similarly, the Hvidoere study group also reported that adolescents with type 1 diabetes watch more than 2 hours of TV daily [23]. In our study, adolescents with type 1 diabetes and non-DM adolescents also exceeded the TV viewing or screen time recommendation of less than 2 hours per day, and A1c and screen time were positively associated in adolescents with type 1 diabetes.

Strengths of this study include a non-DM control group and longitudinal data over a 2-year study period. All questionnaires were interviewer-administered and asked without parent(s) in the room to improve accuracy of data (see Additional file 1: Questionnaires). Limitations of these data include self-reported diet, PA and screen time, therefore reporting bias may exist. The questionnaires used for assessing diet, PA and screen time were not validated. Diet, PA and screen time were not variables associated with the specific aims of the study and there was limited time for lifestyle-related questions. For this reason 3-day food and PA records, a standard for dietary and PA assessment were not used. Diet, PA and screen time questions were created to assess dietary, PA and screen time patterns as recommended by national guidelines (such as daily fruit and vegetable intake and daily PA). Frequency of servings of foods (meals, fruits, vegetables and fried foods) vs. percent or amount of foods consumed was asked to limit the impact of increased energy intake as adolescents get older. The diet, PA and screen time questionnaires were designed to assess lifestyle patterns only and not detailed nutrient intake or direct measures of energy expenditure; however, this would not explain differences reported between groups. We looked at percent of participants who met the PA guidelines in order to compare our findings with the literature and further explain the data. All adolescents were asked how often they consumed sugary beverages on a weekly basis. However, the questionnaire did not differentiate sugary beverages consumed to treat hypoglycemia, although interview-administrators told participants with type 1 diabetes to exclude sugary beverages used to treat hypoglycemia.

Only study participants that completed both study visits were included in this analysis. There were no differences in adolescents with type 1 diabetes for age, sex, A1c, BMI, BMI z-score, BMI percentile, or diabetes duration that completed V1 and V2 compared to adolescents with type 1 diabetes that only completed V1. Among non-DM controls, there were no differences in sex, BMI, BMI z-score, and BMI percentile that completed V1 and V2 compared to non-DM adolescents that only completed V1. Non-DM controls that completed V1 and V2 were significantly younger than those who only completed V1 (14.9 vs. 16.3, p = 0.0023). Although significant, the age difference between non-DM controls who completed V1 vs. V1 and V2 was not substantial (15 years old vs. 16 years old) or clinically relevant. Adolescents with type 1 diabetes reported more non-Hispanic White race/ethnicity which would be expected given that the disease prevalence is higher in the non-Hispanic White race/ethnicity. Factors that may have affected study participants completing V2 include the nature of the age range (adolescents and young adults) and the location of the Barbara Davis Center. The Barbara Davis Center may not be very convenient to visit for the general population since it is a part of a large medical campus, while the adolescents with type 1 diabetes are at the center regularly for their routine clinic visits and their study visit was often scheduled the same day. The teenage and young adult age range is known to be a difficult age to recruit and retain as teenagers become more independent and often leave for college, move away from home and become in charge of their medical care.

## Conclusions

Adolescents with and without type 1 diabetes have similarly poor dietary habits. Over a 2-year period, adolescents with and without type 1 diabetes reported consuming more fruits and vegetables; however, adolescents with type 1 diabetes do not meet the guidelines of daily fruit, vegetable and breakfast intake. Furthermore, fried food intake increased over the 2 year period for adolescents with type 1 diabetes which is of particular concern due to their elevated risk of cardiovascular disease. Adolescents with type 1 diabetes do meet the PA guidelines yet BMI and BMI z-score are higher in adolescents with type 1 diabetes compared to non-DM controls. Screen time was also elevated over the recommended 2 hours or less per day and total screen time increased over time. Adolescents with type 1 diabetes and non-DM controls have similarly poor dietary patterns, despite adolescents with type 1 diabetes receiving dietary education and support through their diabetes care. Some diet and PA improvements were seen in adolescents with type 1 diabetes over a 2-year period. Therefore, adolescence could be a beneficial time to target diet and lifestyle interventions to take advantage of this time period when behaviors are being modified.

## Competing interests

The authors declare that they have no competing interests.

## Authors’ contributions

FKB coordinated and supervised the data collection, wrote the manuscript and approved the final manuscript for submission. RPW conceptualized and designed the study, researched, contributed to discussion, reviewed and revised the manuscript and approved the final manuscript for submission. JSB reviewed and revised the manuscript and approved the final manuscript for submission. NN analyzed data and reviewed and revised the manuscript and approved the final manuscript for submission. DMM researched, analyzed data, contributed to the discussion and reviewed and revised the manuscript and approved the final manuscript for submission. All authors read and approved the final manuscript.

## Supplementary Material

Additional file 1 Interviewer-administered questionnaires. Click here for file
