# Supplementary material for: Changes in diet and physical activity in adolescents with and without type 1 diabetes over time
Source: Int J Pediatr Endocrinol. 2014 Aug 15;2014(1):17. doi: 10.1186/1687-9856-2014-17 (PMC4154618; doi:10.1186/1687-9856-2014-17)
Supplement: Additional file 1 — Interviewer-administered questionnaires. [file 1687-9856-2014-17-S1.doc]

COMIRB #:07-0780

Study ID#:

**Exercise:** (*adolescent to answer)*

Participates in sports/physical activity: Yes No

*(Such as PE class, walking, biking, playing outside, sports, dance etc)*

Type of sport/physical activity: ____________________________________________

Days/Week: 1 2 3 4 5 6 7

Hours/Day: .

Exercise Level: light moderate hard *(read from table)*

TV/Day: . hours *(average; subject says 1-2 hrs pt 1.5)*

Computer/Day: . hours

Video/Electronic games/Day: . hours

During the last week, subject was: less active than usual

about as active as usual

more active than usual

*Interviewer: Use these examples when describing what light, moderate, and hard exercise levels:*

_______________________________________________

| **Level of Intensity** | **RPE** | **Physical Cues** |
| --- | --- | --- |
| Light | Easy | Does not induce sweating unless it's a hot, humid day. There is no noticeable change in breathing patterns. |
| Moderate | Somewhat hard | Will break a sweat after performing the activity for about 10 minutes. Breathing becomes deeper and more frequent. You can carry on a conversation but not sing. |
| High | Hard | Will break a sweat after 3-5 minutes. Breathing is deep and rapid. You can only talk in short phrases |

**Tobacco Use:** (*adolescent to answer)*

1. Do you live with anyone who currently smokes cigarettes or use tobacco products?

1 □yes 2 □no 3 □Not applicable (lives alone)

1. Do people smoke or use other tobacco products in the area where you work?

1 □yes 2 □no 3 □Not applicable (Not employed)

1. How many of your friends smoke or use other tobacco products?

0 □None 1 □ Less than half 2 □ Half or more than half

3 □ Almost all my friends

1. Have you ever smoked cigarettes or use tobacco products (e.g. chewing tobacco (‘chew, dip, snuff’…), pipes, cigars, (other??)?

1 □Yes 2 □No (If No, go to Alcohol questionnaire)

1. Do you smoke cigarettes or use other tobacco products now?

1 □Yes 2 □No

5a. If Yes, what type of tobacco products do you use?

- - 1. □Cigarettes
    2. □Cigars
    3. □Chewing tobacco
    4. □Other (list):_________________

1. □Unknown/Undisclosed
2. How long have you been smoking cigarettes/using tobacco products?

. **Months**

*(Interviewer: if subject answers in years or days calculate into months)*

1. How many cigarettes do you currently smoke (or amount of other tobacco products used if applicable)? (1 pack = 20 cigarettes) *(round to nearest whole #)*

7a. Yesterday? _______________

7b. In the last week? _______________

7c. In the last month? _______________

**Alcohol Use:** (*adolescent to answer)*

1. How many of your friends drink alcohol?

0 □None 1 □ Less than half 2 □ Half or more than half

3 □ Almost all my friends

1. Have you ever drank alcohol?

1 □Yes 2 □No (If No, end questionnaire here)

1. Do you drink alcohol now? 1 □yes 2 □no
2. How often do you drink alcohol?

1 □Daily 2 □Weekly 3 □Monthly 4 □Yearly

5 □Other: (church, with parents etc) _____________________________

1. How long have you been drinking alcohol? . **Months**

*(Interviewer: if subject answers in years or days calculate into months)*

**Dietary Practices:** (*adolescent to answer)*

1. How often do you eat at restaurants (including fast food)?

0□. 1 time per month or less 1□ 2-3 times per month 2□ 1-2 times per week

3□. 3-4 times per week 4□. 5-6 times per week 5□. Every day

1. How often do you drink sweetened beverages (juice or juice containing drinks, soda, energy drinks, sports drinks, other?)?

0□. 1 time per month or less 1□ 2-3 times per month 2□ 1-2 times per week

3□. 3-4 times per week 4□. 5-6 times per week 5□. Every day

1. How many meals a day do you eat?

1□ One 2□. Two 3□. Three 4□. Four 5□. Five or more

1. How many snacks a day to you eat?

1□ One 2□. Two 3□. Three 4□. Four 5□. Five or more

1. How often do you eat breakfast?

0□. 1 time per month or less 1□ 2-3 times per month 2□ 1-2 times per week

3□. 3-4 times per week 4□. 5-6 times per week 5□. Every day

1. How often do you eat fruit?

0□. 1 time per month or less 1□ 2-3 times per month 2□ 1-2 times per week

3□. 3-4 times per week 4□. 5-6 times per week 5□. Every day

1. How often do you eat vegetables?

0□. 1 time per month or less 1□ 2-3 times per month 2□ 1-2 times per week

3□. 3-4 times per week 4□. 5-6 times per week 5□. Every day

1. How often do you eat fried foods (such as french fries, chicken nuggets, fried chicken, chips etc)?

0□. 1 time per month or less 1□ 2-3 times per month 2□ 1-2 times per week

3□. 3-4 times per week 4□. 5-6 times per week 5□. Every day

1. How often do you eat desserts/sweets (such as cookies, cakes, doughnuts, candy etc.)?

0□. 1 time per month or less 1□ 2-3 times per month 2□ 1-2 times per week

3□. 3-4 times per week 4□. 5-6 times per week 5□. Every day
